# Intravitreal Bevacizumab for Choroidal Metastasis of Lung Carcinoma; a Case Report

**Published:** 2010-10

**Authors:** Aída Sánchez de la Barquera Cordero, Rene Alfredo Cano Hidalgo

**Affiliations:** Instituto Oftalmológico Hospital Conde de Valenciana, México City, Mexico

**Keywords:** Antiangiogenic, Antineoplasic, Bevacizumab, Choroidal Metastasis, Lung Carcinoma, Ocular Tumors

## Abstract

**Purpose:**

To report an alternative treatment for metastatic tumors within the eye.

**Case Report:**

Five intravitreal injections of bevacizumab (2.5 mg each) were performed in one eye of a 50-year-old woman with choroidal metastasis of lung carcinoma. Tumor size was reduced, pain disappeared, vision improved, and there were no secondary reactions. Vision improved from 20/40 to 20/20 and metamorphopsia decreased. Ten months after initiating treatment, an ultrasonographic study revealed no residual tumor, the choroid was normal in thickness and fluorescein angiography revealed a scar but no mass lesion.

**Conclusion:**

Intravitreal bevacizumab displayed beneficial effects in reducing tumor size and improving symptoms in this case of choroidal metastasis of lung carcinoma. The antineoplasic properties of this agent make it a viable alternative for treatment of metastatic choroidal tumors.

## INTRODUCTION

Metastatic choroidal tumors are the most common intraocular malignancy in adults of whom 50% to 70% are women. In female subjects, the primary tumor is in the breast in 70% to 80% of cases while lung cancer accounts for another 10%. In male subjects, lung cancer account for 40% to 60% of cases.^1^,^2^

Treatment for choroidal metastasis is based on the degree of activity and location of the primary tumor. If the patient is under systemic chemotherapy and the ocular tumor is asymptomatic, no ocular treatment is indicated. Treatment is recommended if the metastasis is threatening vision or the globe despite chemotherapy. In such cases, external beam radiation therapy or plaque radiotherapy and proton beam irradiation have been recommended.^2^,^3^

Bevacizumab is a humanized recombinant monoclonal antibody which targets the vascular endothelial growth factor (VEGF). VEGF is expressed in high levels in certain tumors. In 2004, bevacizumab was approved by the US Food and Drug Administration for metastatic colon cancer, and two years later for use in lung cancer.^4^ In ophthalmology, it has been widely used as an intravitreal agent for treatment of proliferative (neovascular) eye diseases.^5^,^6^

There have been a number of reports on anti-angiogenic therapy for ocular tumors.^7^,^8^ Intraocular bevacizumab has also been used in osteomas with neovascular membranes.^9^ Due to these reports and the fact that there are limited treatment options, we considered the use of bevacizumab as a valid alternative.

Herein we present a patient with metastatic choroidal carcinoma originating from the lung who received treatment with intravitreal bevacizumab (IVB) resulting in significant subjective and objective improvement.

## CASE REPORT

A 50-year-old woman with history of non-small cell lung carcinoma since November 2005, had been receiving chemotherapy with carboplatin, paclitaxel and systemic bevacizumab for 8 months. She was referred on February 2007, due to loss of vision, accompanied with pain and metamorphopsia in her right eye.

**Figure 1:**
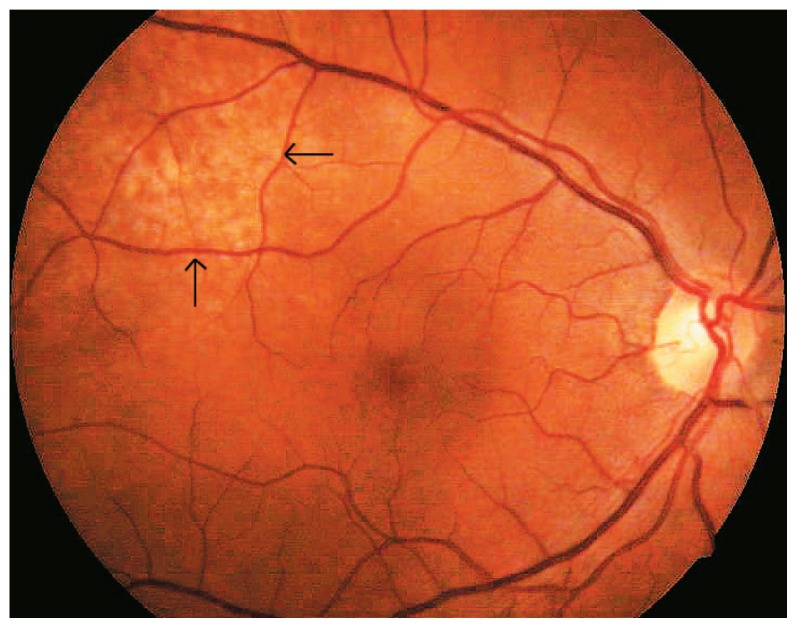
Fundus appearance of the right eye before treatment (February 27, 2007); a solid subretinal tumor can be observed superior and temporal to the fovea (arrows).

**Figure 2:**
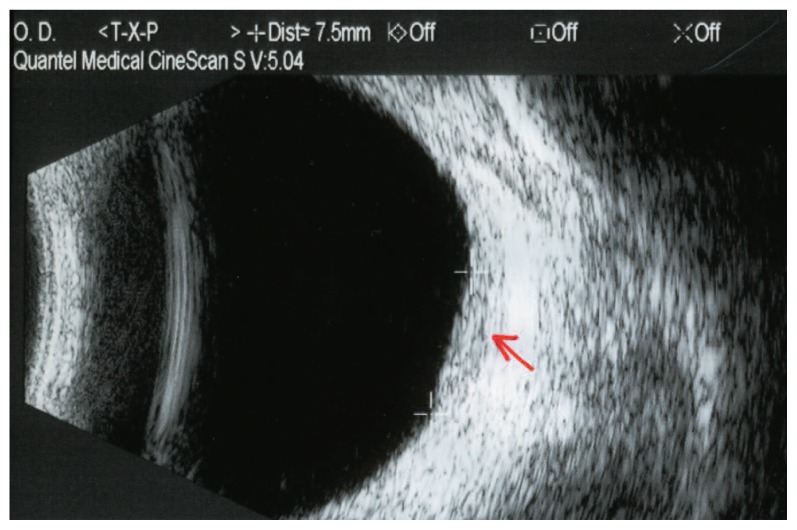
Ultrasound scan of the same eye as in figure 1 before treatment (March 9, 2007); a choroidal mass 8.8×7.5 mm in basal dimensions and with maximal elevation of 1.74 mm is apparent in the temporal aspect of the globe (arrow).

**Figure 3:**
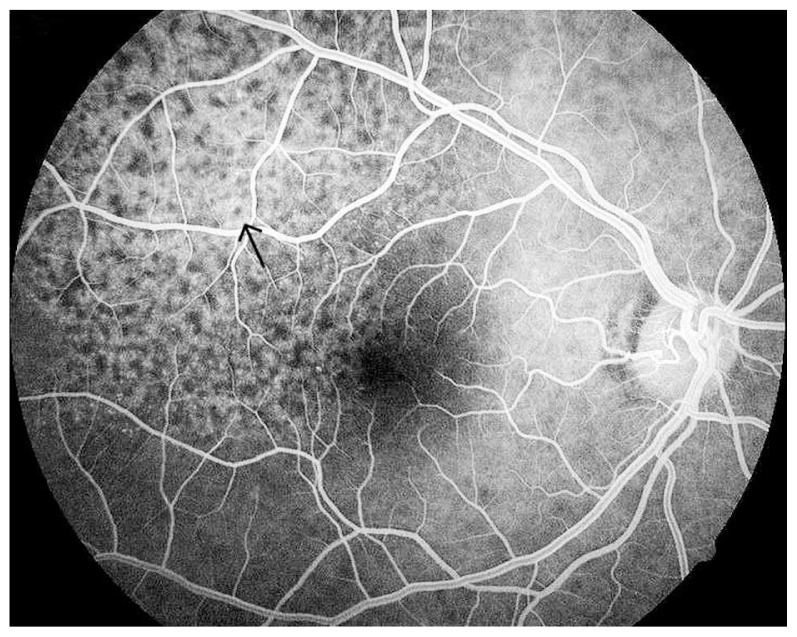
Pretreatment angiography; a hyperfluorescent non-vascularized tumor can be seen as a mottled pattern without leakage (arrow).

Vision was 20/40 in her right and 20/20 in her left eye. Ophthalmoscopy revealed a subretinal mass, light orange in color approximately four disc diameters in size, temporal and superior to the fovea, without retinal detachment in the right eye (Fig. 1). Ultrasonography demonstrated a choroidal mass 8.8×7.5 mm at its base with maximal elevation of 1.74 mm in the right eye (Fig. 2). Fluorescein angiography showed a mottled pattern in the region of the tumor without leakage (Fig. 3).

**Figure 4:**
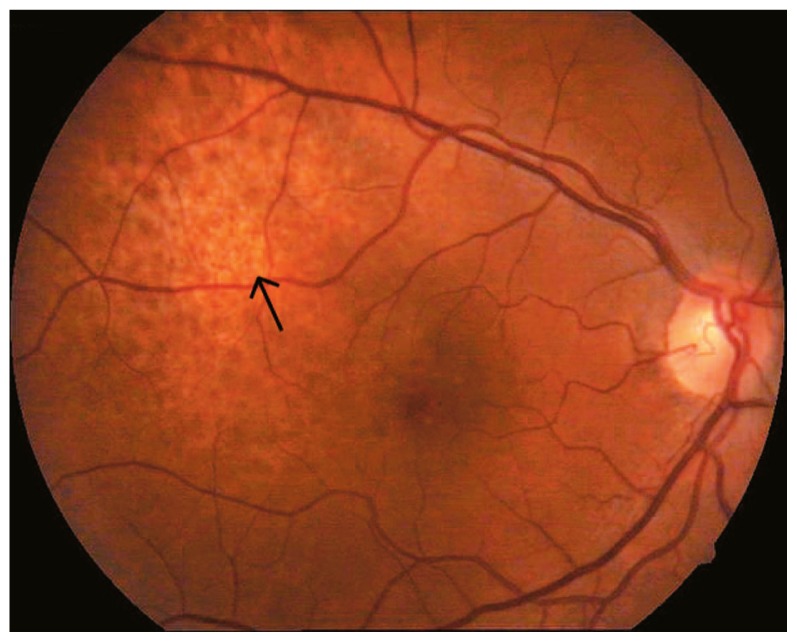
Post-treatment fundus image (March 6, 2008); the tumor demonstrates less projection and more pigmentation than before, without retinal detachment (arrow).

**Figure 5:**
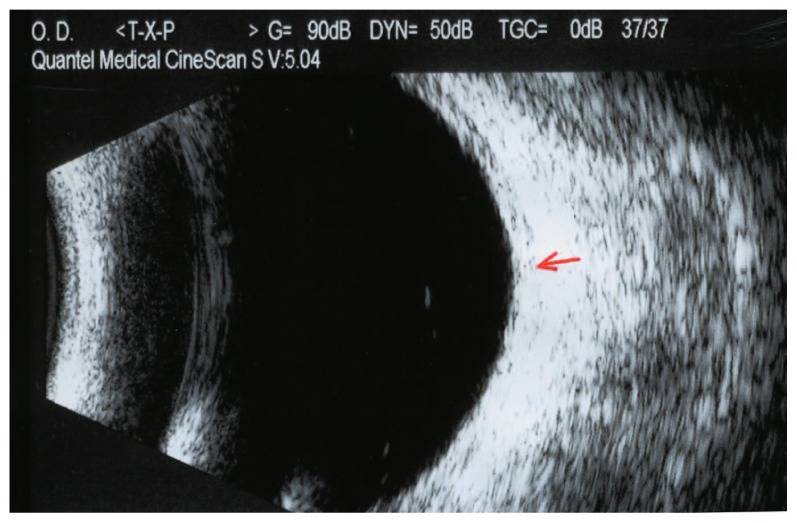
Post-treatment ultrasound scan (January 9, 2008); no mass is detectable and the choroid reveals normal thickness (arrow).

**Figure 6:**
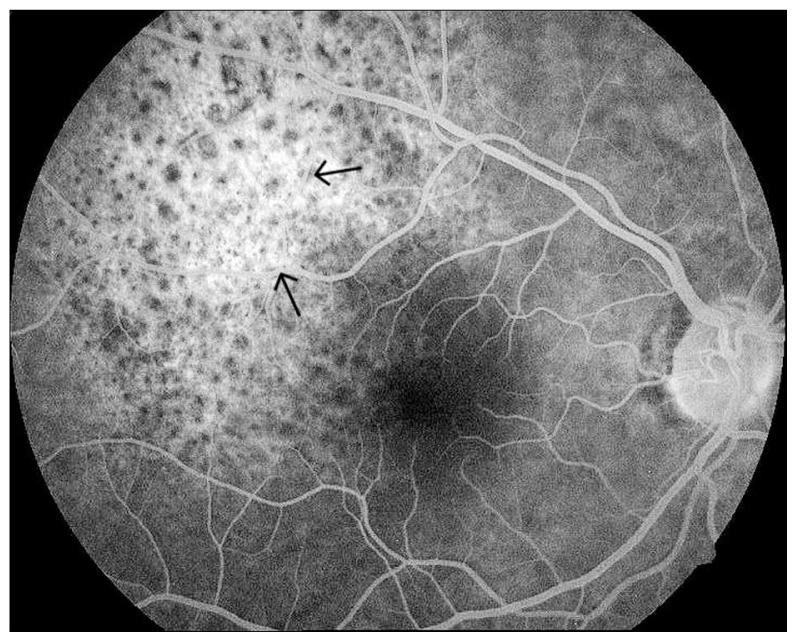
Post-treatment angiography (March, 2008). The tumor appears less hyperfluorescent. The same mottled pattern can be seen without leakage (arrows).

We injected 2.5 mg bevacizumab intravitreally in the right eye of the patient five times over a period of seven months. Table 1 shows the date of each injection. Pain disappeared after the third injection, metamorphopsia decreased and vision improved to 20/20. Fundus examination revealed regression of the tumor (Fig. 4). On January 2008, a B-scan was performed with special attention to the temporal aspect of the globe and no tumor could be detected; in addition, the choroid was of normal thickness (Fig. 5). The angiogram showed a less hyperfluorescent plaque without leakage (Fig. 6).

## DISCUSSION

Bevacizumab was initially developed to treat colon cancer due to its main action as an inhibitor of angiogenesis for newly formed tumor vessels. We used it in our patient, bearing this fact in mind. In our patient systemic chemotherapy was inadequate to control the ocular complications. Additional therapy with intraocular bevacizumab caused the tumor to contract and disappear together with resolution of pain. We believe that intravitreal bevacizumab acted directly on the metastasis.

There are some reports on this treatment modality describing favorable outcomes and they recommend this approach as a therapeutic alternative. Kwo et al^10^ reported a patient treated with IVB for choroidal metastasis of colorectal carcinoma. In other reports where the tumors had been benign, vision improved due to resolution of the accompanying serous retinal detachment.^8^,^9^

More cases with further follow-up and controlled trials are necessary to determine the long-term effects of IVB for this particular indication.

## Figures and Tables

**Table 1:** Dates of bevacizumab injections

Injection	Date (month/day/year)
No. 1	03/07/07
No. 2	04/11/07
No. 3	06/11/07
No. 4	08/15/07
No. 5	10/17/07
